# Electronic and Magnetic Properties of Ni-Doped Zinc-Blende ZnO: A First-Principles Study

**DOI:** 10.3390/nano8050281

**Published:** 2018-04-26

**Authors:** Suqin Xue, Fuchun Zhang, Shuili Zhang, Xiaoyang Wang, Tingting Shao

**Affiliations:** 1 College of Mathematics and Computer Science, Yan’an University, Yan’an 716000, Shanxi, China; ydxsq@yau.edu.cn; 2 College of Physics and Electronic Information, Yan’an University, Yan’an 716000, Shanxi, China; zhangshuili74@163.com (S.Z.); yettawang15@163.com (X.W.); retastt@126.com (T.S.)

**Keywords:** Ni-doped, ZnO, first-principles, ferromagnetic and anti-ferromagnetic

## Abstract

The electronic structure, band structure, density of state, and magnetic properties of Ni-doped zinc-blende (ZB) ZnO are studied by using the first-principles method based on the spin-polarized density-functional theory. The calculated results show that Ni atoms can induce a stable ferromagnetic (FM) ground state in Ni-doped ZB ZnO. The magnetic moments mainly originate from the unpaired Ni 3*d* orbitals, and the O 2*p* orbitals contribute a little to the magnetic moments. The magnetic moment of a supercell including a single Ni atom is 0.79 μ_B_. The electronic structure shows that Ni-doped ZB ZnO is a half-metallic FM material. The strong spin-orbit coupling appears near the Fermi level and shows obvious asymmetry for spin-up and spin-down density of state, which indicates a significant hybrid effects from the Ni 3*d* and O 2*p* states. However, the coupling of the anti-ferromagnetic (AFM) state show metallic characteristic, the spin-up and spin-down energy levels pass through the Fermi surface. The magnetic moment of a single Ni atom is 0.74 μ_B_. Moreover, the results show that the Ni 3*d* and O 2*p* states have a strong *p*-*d* hybridization effect near the Fermi level and obtain a high stability. The above theoretical results demonstrate that Ni-doped zinc blende ZnO can be considered as a potential half-metal FM material and dilute magnetic semiconductors.

## 1. Introduction

Diluted magnetic semiconductors (DMSs) are a new type of semiconductor material which obtain FM properties by doping 3*d* transition-metal atoms. They have been investigated extensively because of their potential usage of both the charge and spin properties of freedom of carriers in spintronic devices. Many researchers expected to be able to prepare ultra-high speed, super bandwidth, low-power, and non-volatile spintronic devices based on DMSs, such as spin field effect transistors (Spin-FET), spin light-emitting diodes (Spin-LED), spin resonant tunneling diodes (Spin-RTD), optical isolators, magnetic sensors, nonvolatile memory cells, etc., [1,2,3,4,5,6,7,8]. During the past years, the investigation into the applications of ZnO nano-semiconductor materials has become a hot topic in the wide-gap semiconductor materials field [9,10]. ZnO materials have n-type semiconductor characteristics due to the inherent oxygen vacancy and other intrinsic n-type defects, because of this, ZnO materials are potential DMSs materials with a high Curie temperature. Sato et al. [11] have researched transition-metal atoms (Mn, V, Cr, Fe, Co, Ni) doped ZnO materials through first-principle calculations, and the results show that the doping system has FM properties. Ueda et al. [12] have also successfully prepared 3*d* transition-metal-doped ZnO thin film materials, the results show that the Curie temperature is greater than 280 K. Herein, we especially highlight Igamberdiev’s work [13], providing and reporting the magnetic phase transitions in ZnO doped with Mn for DMSs. Liu et al. [14] have predicted the possibility of ferromagnetism in wide-band gap semiconductors ZnO and GaN based on the recent progress in the theoretical and experimental studies of ZnO- and GaN-based DMSs. In terms of Ni-doped ZnO research, Zhao has reported room temperature FM in Zn_1−*x*_Ni*_x_*O (*x* = 0.1%, 0.4%, 0.7%, 1.0%) powders using the sol-gel technique [15]. Liu et al. [16] have successfully synthesized Ni-doped ZnO with pulsed laser deposition (PLD), the results show that Ni-doped ZnO has FM properties. Cui et al. [17] have synthesized Ni- and Co-doped ZnO nanowire arrays with an electrochemical method at 90 °C, and the results show that magnetic nanowires have anisotropic FM properties. Wang et al. [18] have prepared Ni-doped ZnO nanowire arrays by means of metal vapor vacuum arc (MEVVA) ion source doping technology, and the results show that the electron transport ability increases by 30 times and the absorption peak exhibits red-shift phenomena. Wakano et al. [19] have also synthesized Ni-doped ZnO thin film materials, and the results show that FM features appear at 2K, while PM properties appear at 300K. Al-Harbihas synthesized a Zn_1−*x*_Ni*_x_*O nanorod with excellent UV emissive power by means of a low-temperature hydrothermal method [20]. Cheng et al. have also prepared a Zn_1−*x*_Ni*_x_*O nanorod with room temperature FM features by means of a low-temperature hydrothermal method [21]. However, Yin et al. have reported that no FM feature has been observed in Zn_1−*x*_Ni*_x_*O nano-materials [22].

Until now, most studies have been focused on 3*d* transition-metal-doped wurtzite ZnO materials, including synthesis, theoretical prediction, and the FM coupling mechanism of DMSs at room temperature. However, theoretical or experimental study on transition-metal atom-doped ZB ZnO DMSs is uncommon. Most research studies show that the samples prepared with transition-metal-doped ZnO materials vary greatly in performance, being poor in experiment repeatability. Meanwhile, the magnetic origin and magnetic coupling mechanism should also be further studied.

In this paper, we replace the two nearest neighboring (Ni–O–Ni) and the next nearest neighboring (Ni–O–Zi–O–Ni) Zn atoms in different positions with two Ni atoms. The purpose of our work is to study the electronic structures, band structure, density of state, FM and AFM properties, and magnetic coupling mode of Ni-doped ZB ZnO using the density functional theory, and reveal the magnetic origin and FM coupling mechanism. The research results will provide a theoretical basis for the application of ZnO DMSs.

## 2. Theoretical Models and Calculated Methods

**Figure 1:**
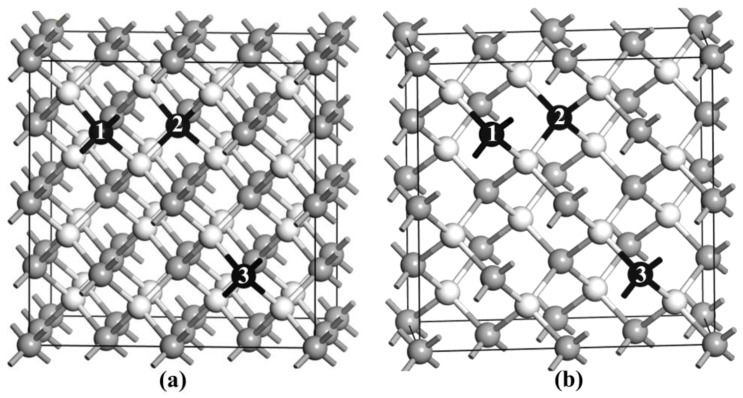
The crystal model of Ni-doped zinc-blende (ZB) ZnO (the gray and white balls indicate Zn and O atoms, respectively). The positions of Zn substituted by Ni are denoted by 1 and 2–3, (**a**) 2 × 2 × 2 supercell; (**b**) 1 × 2 × 2 supercell.

To study the electronic structure and the magnetism of Ni-doped ZB ZnO, we constructed the model of Ni-doped ZB ZnO [23], as shown in Figure 1a. The doping configurations of Ni-doped ZB ZnO are based on the 2 × 2 × 2 ZB supercell containing 32 Zn atoms and 32 O atoms with a doping concentration of 6.25 at. %. In order to study the stability of coupling magnetic interactions in the ground state, two different spatial constructs (refered to as configuration I (1,2) and II (1,3)) based on the Ni substitution sites were studied, where the two nearest-neighboring or next nearest-neighboring Zn atoms were replaced by two Ni atoms. In configuration I, the two neighboring Ni atoms were direct coupling through a single O (Ni–O–Ni) atom at the minimum distance. In configuration II, The two neighboring Ni atoms were indirect coupling through an atomic chain including two O atoms and one Zn atoms (Ni–O–Zn–O–Ni) at a comparatively larger distance. In addition, in order to confirm the suitability of the employed supercells, we also constructed a 1 × 2 × 2 supercell containing 16 Zn atoms and 16 O atoms (Figure 1b); the model adopted the same doping method as the 2 × 2 × 2 ZB supercell. These two different spatial constructs (refered to as configuration III (1,2) and IV (1,3)) had a corresponding doping concentration of 12.5 at. %.

First-principles calculations based on the density functional theory (DFT) [24] with a plane-wave pseudo-potential basis were performed using the VASP software package [25] to study Ni-doped ZB ZnO. The detailed parameters settings are shown as follows: the pseudo potential is treated by using projector augmented waves (PAW) potentials [26] to represent the interaction between electron and ionic. The electron exchange-correlation potential is performed by using the Perdew-Burke-Ernzerhof (PBE) formulation of Generalized Gradient Approximation (GGA). The valence electron configurations of O, Zn and Ni atoms were performed as 2*s*^2^2*p*^4^, 3*d*^10^4*s*^2^ and 3*d*^8^4*s*^2^, respectively. The cutoff energy of the plane-wave basis set was set at 420 eV. The self-convergence accuracy of the iterative process was set as less than 1 × 10^−6^ eV/atom. The maximal displacement convergence was 1 × 10^−4^ nm and the internal stress was less than 0.1 GPa. For the Brillouin-zone integration, the 6 × 6 × 6 *k*-point grids for the 64 atoms were employed by using a special *k*-points sampling scheme of Monkhorst-Pack in the reciprocal space.

## 3. Results and Discussion

Table 1 shows the lattice parameters of Ni-doped ZB of the 2 × 2 × 2 and 1 × 2 × 2 supercells. For configuration I and configuration II, the lattice parameters a, b, and c are significantly higher than that of pure ZnO, indicating that the volume of Ni-doped ZB ZnO expands slightly. This phenomenon cannot be explained by conventional theories, because the Ni^2+^ ion radius (0.69 Å) is smaller than the Zn^2+^ ion radius (0.74 Å). We believe that there is no new compound formed after doping Ni^2+^ ion, but the replacement of Zn^2+^ with Ni^2+^ causes lattice distortion. As the Ni^2+^ ion radius is smaller than the Zn^2+^ ion radius, the residual stress generated during the crystallization process results in an increased mutual repulsion between the Ni^2+^ ion polarization charges, the system energy rises and so triggers the volume increase. The observed expansion in volume *V*_0_ has also been found in nanoplatelets of Ni-doped ZnO [27] and Ni- and Co-doped ZnO [28]. Moreover, for configuration III and configuration IV, the calculated lattice constants showed a good consistency with the 2 × 2 × 2 supercell, and four configurations of Ni-doped ZB ZnO were approximate to the experimental value [23], the calculated relative error was less than 2%. In order to satisfy the limit of doping concentration in experimental results, we adopted configuration I and configuration II to investigate the electronic and magnetic properties with a doping concentration of 6.25 at. %.

Table 2 shows the FM, AFM coupled configuration, bond length, and magnetic moments of Ni-doped ZB ZnO; Zn–O and Ni–O, respectively, are the neighbor bond lengths of Zn. In order to determine the ground state of Ni-doped ZB ZnO, the total energy and energy differences of the FM and AFM phases in the above configurations were calculated. The Δ*E* is the energy difference (Δ*E = E_AFM_ − E_FM_*) of the AFM and FM states in relaxation. If the Δ*E* is smaller than zero, it indicates that the AFM state is more stable, if the Δ*E* is larger than zero, it indicates that the FM state is more stable. The total energies for configuration I and II are calculated as −68055.9 eV and −68055.5 eV, respectively. The comparatively lower energy in configuration I indicated that Ni-doped ZB ZnO have a tendency to cluster together and favor short-range Ni–Ni magnetic coupling, so the doping system is more likely to form impurity phases like NiO*_x_*. The observed phenomenon has also been found in other Ni-doped systems [29,30].

As shown in Table 2, we compared Zn_30_Ni_2_O_32_ with pure ZB ZnO, the total energy of the two configurations increased after being doped with Ni atoms, indicating that the stability of the crystal structure decreased. For configuration I, the energy of the FM state was 0.5 eV lower than the AFM state. While for configuration II, the energy of the FM state was 0.2 eV lower than the energy of the AFM state. The above calculation results show that the ground state of the two configurations of Ni-doped ZB ZnO corresponds to a FM state. In particular, it is obvious that the energy differences (Δ*E*) increase with the decrease in the length of the Ni–O bonds. The largest energy difference (Δ*E*) is 0.5 eV corresponding to configuration I. The above results imply that a room temperature ferromagnetism of Ni-doped ZnO can be expected. In addition, the Ni–O and Zn–O bond lengths of Ni-doped ZB ZnO increased significantly more than the Zn–O bond lengths of pure ZB ZnO, which is primarily due to the geometry optimization lattice distortion caused by Ni atoms doping. The chemical interaction between Ni and ZnO was studied using calculated Mulliken bond populations (Table 2), because bond population can be used to analyze the ionic or covalent bonds [31]. The results indicated that the covalent bond of the doping system became stronger and the ionic bond became weaker than those of pure ZB ZnO with the increasing value of Mulliken bond populations; the covalent bond of FM state of configuration I was strongest. We also see in Table 2 that the magnetic moments of Ni atoms were 0.77 μ_B_ and −0.77 μ_B_ for AFM coupling in configuration I, while the magnetic moment of Ni atoms was 0.79 μ_B_ for FM coupling, but the calculated magnetic moments were less than the theoretical magnetic moment of 2.0 μ_B_. The results show that Ni 3*d* states lost electrons at a greater rate; O atoms around two Ni atoms also induced a weak magnetic moment, the magnetic moments were 0.05 μ_B_ and 0.11 μ_B_. For configuration II, the magnetic moments of the FM and AFM state increased slightly, and the Ni atom and neighbor O atom had weak magnetic moments. The above results indicate that Ni-doped ZB ZnO has a certain magnetism, which is mainly because the Ni 3*d* state in outer valence electron configuration is 3*d*^8^; the Ni^2+^ atom does not introduce extra valence electrons, but Ni 3*d* orbits form a Ni^2+^–O^2−^–Ni^2+^ spin polarization with neighboring O atoms, thus leading to the outer valence electron redistribution.

**Figure 2:**
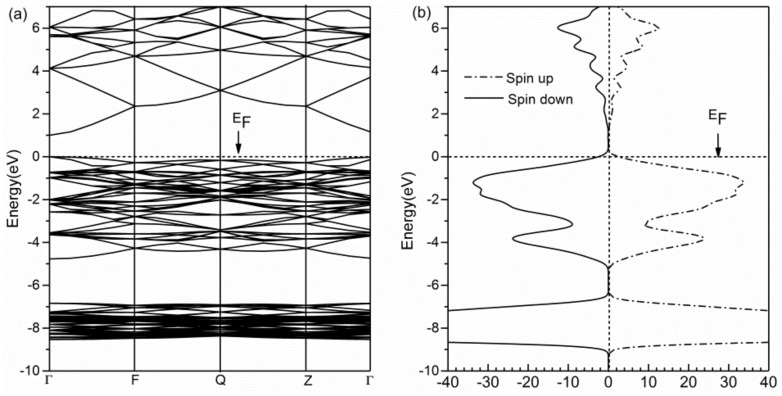
(**a**) Band structure of pure ZB ZnO; (**b**) Total DOS of pure ZB ZnO.

For comparing and analyzing the influence of the electronic structure and magnetic properties of Ni-doped ZB ZnO, we calculated the band structure and density of states (DOS) of pure ZnO. As can be seen from Figure 2a,b, the bottom of the conduction band and top of the valence band of pure ZnO are located at Γ point of the Brillouin zone, the calculated band gap is 1.0 eV, and showed ZB ZnO is a direct wide band-gap semiconductor material, consistent with the existing theoretical results [23,32]. The total DOS of pure ZnO shows that the spin DOS is completely symmetrical, which indicates that pure ZnO is not magnetic.

**Figure 3:**
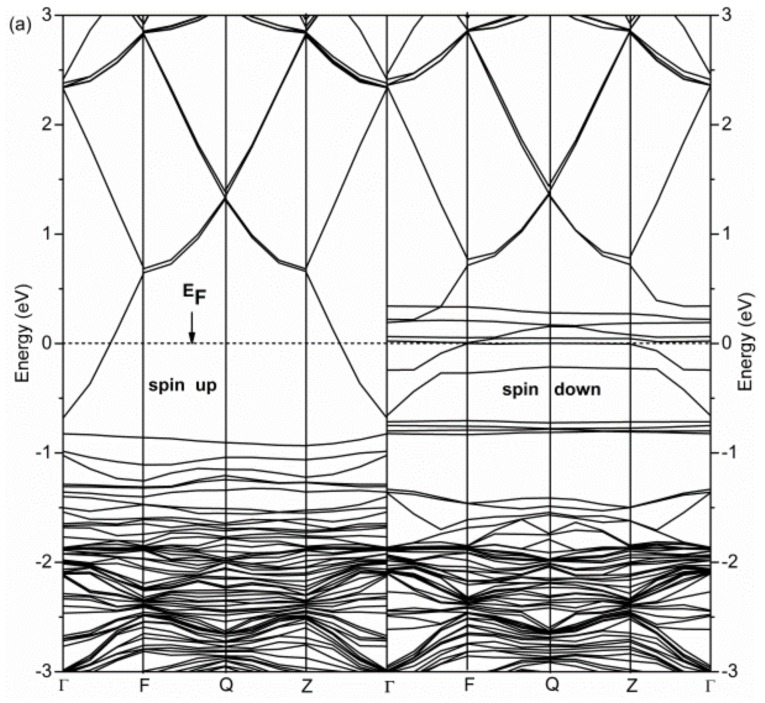

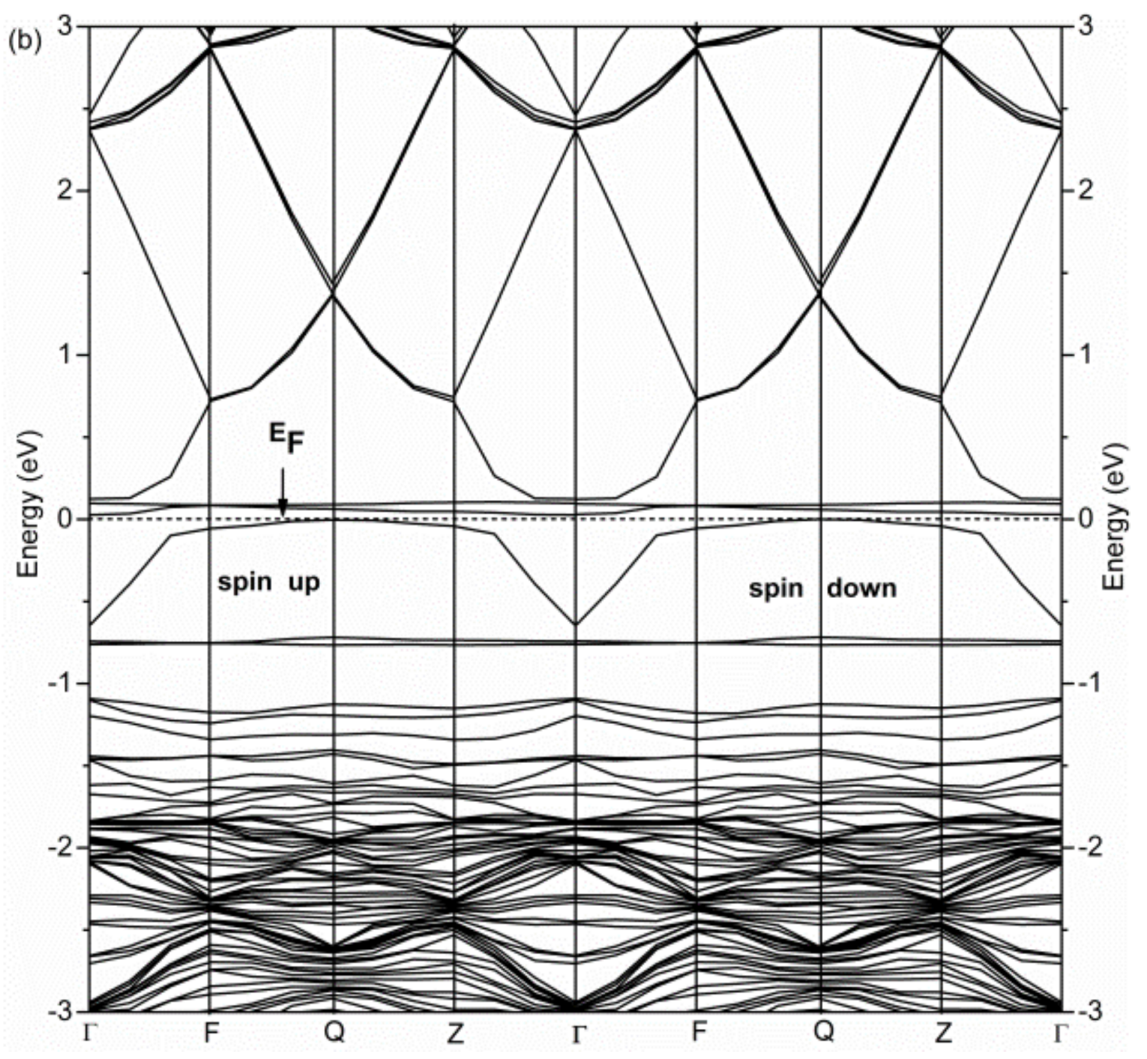
Band structures of Ni-doped ZB ZnO. (**a**) FM; (**b**) AFM.

In order to study the mechanism of magnetic coupling, the calculated band structure of Ni-doped ZnO will be discussed in Figure 3; this is the band structure of the FM and AFM state for Ni-doped ZB ZnO. As can be seen from the Figure 3b, the energy levels of spin-up and spin-down are almost the same, the magnetic moment of the AFM state is zero, and the doped system is not magnetic. Moreover, the spin energy levels pass through the Fermi surface, the results exhibit metallic characteristics for the AFM state. In addition, at the top of the valence band, there appeared two and a half filled impurity levels. These impurity levels, which are important for carrier mobility, which is conducive to Ni-doped ZnO carrier transport, can significantly improve the Ni-doped ZnO conductive properties. For the band structure of the FM state, it can be seen from Figure 3a, the energy levels of spin-up and spin-down are obviously different in the band structures, the energy level of spin-down electrons has undergone an obvious spin splitting at the Fermi level, and exhibits a strong spin polarization phenomenon. The band structures have asymmetric distribution characteristics, the doped systems show magnetic semiconductor properties. Especially in the Fermi surface, Ni atoms and neighboring O atoms have strong hybrid interactions, and crystal field effects lead to strong hybrid orbitals splitting, the spin-down energy levels pass through the Fermi surface, and the material appears to be in a semi-metallic FM state. The results indicate that Ni-doped ZB ZnO is a good half-metallic magnetic material.

**Figure 4:**
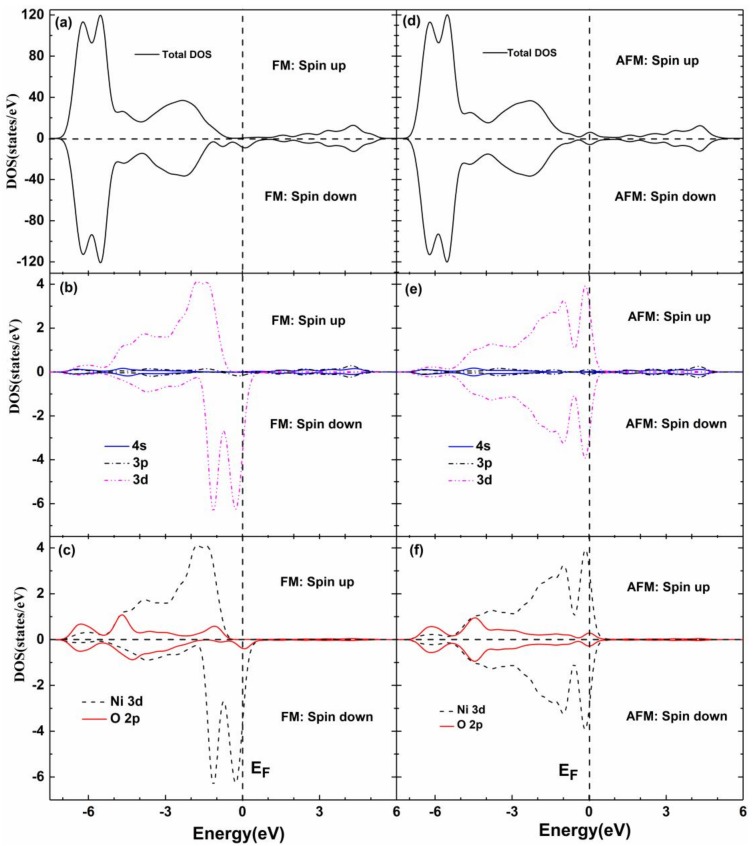
Total and partial DOS of Ni-doped ZB ZnO. (**a**) total DOS of FM; (**b**) partial DOS of Ni atoms for FM; (**c**) partial DOS of Ni 3*d* and O 2*p* for FM; (**d**) total DOS of AFM; (**e**) partial DOS of Ni atoms for AFM; (**f**) partial DOS of Ni 3*d* and O 2*p* for AFM.

To further explain the magnetic properties of Ni-doped ZB ZnO, we calculated the total and partial DOS of the FM and AFM states, as shown in Figure 4. As can be seen from Figure 4a, the total and partial DOS are obviously spin-orbit splitting near the Fermi level; the spin-down DOSs of the Ni 3*d* state at the Fermi level split into two peaks, the spin-up and spin-down DOS underwent a significant shift, there is a clear spin splitting phenomenon. Near the Fermi surface, the spin-up DOS had semiconductor properties, while the spin-down electrons had a metallic property, and it is obvious that the FM system is caused by the 100% electron-spin polarization and the half-metallic magnetic material. This is mainly due to the Ni 3*d*^8^ orbital electrons between the atoms, forming Ni^2+^–O^2−^–Ni^2+^ orbital coupling, resulting in the outermost valence electrons atomic migration. Especially in the neighboring layer, the partially occupied spin-down Ni 3*d* orbital electrons are likely to stabilize the FM state by filling the lower bonding state, and electrons in the Ni 3*d* state are arranged to form a FM coupling in the same direction. As seen in Figure 4e,f, the DOS of the AFM state is symmetrical, the upper valence band (–3 eV–0.0 eV) is mainly composed of Ni 3*d* and O 2*p* states with a strong hybridization of orbitals, causing the Ni 3*d* and O 2*p* states to move in a higher energy direction and through the Fermi surface. Therefore, the doped system of the AFM state mainly forms metallic bonds by Ni 3*d* and O 2*p* orbital hybridization.

**Figure 5:**
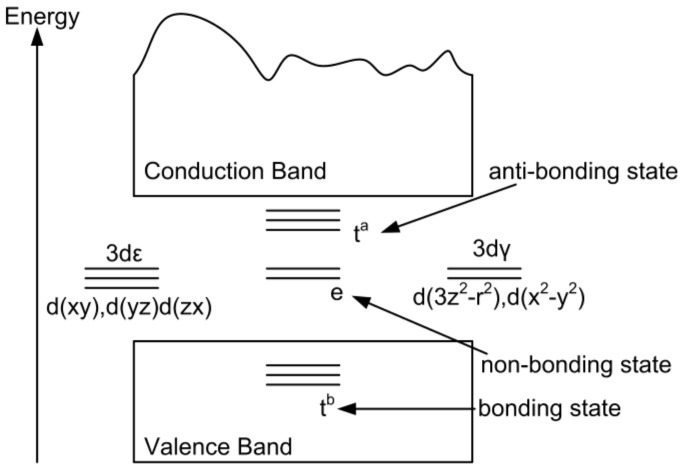
Energy graph of 3*d* transition-metal atoms in a tetrahedral crystal field (*T_d_*).

To further investigate the coupling mechanism of Ni-doped ZB ZnO, we approximated the Ni atom in a tetrahedral crystal field (*T_d_*), the specific structure is shown in Figure 5. Where the five-fold degeneracy of the Ni 3*d* band was transformed to a three-fold degenerate *t*_2*g*_ state and two-fold degenerate *e_g_* states. In accordance with the Hund rule and double exchange mechanism, the calculated results show that a coupling model appeared in the FM and AFM states, when we replaced Zn^2+^ ions with Ni^2+^ ions and formed partially occupied *t*_2*g*_ orbital electron. This is the lowest energy state corresponding to the FM state, since four electrons occupy *t*_2*g*_ orbits, four electrons occupy *e_g_* orbits. As a result spin electrons of the Ni^2+^ *e_g_* state and neighboring Ni^2+^ *e_g_* states are in the same direction, and Ni^2+^ ions can be coupled to each other and moved between the two neighboring Ni^2+^ ions, resulting in Ni-doped ZB ZnO appearing in a stable FM state. The conclusion is consistent with our above-calculated results.

## 4. Conclusions

In this study, the electronic structure and coupling mechanism of Ni-doped ZB ZnO are investigated by using the spin polarization approach based on first-principles DFT. By analyzing the energy, magnetic, and electrical properties of different magnetic coupling models, we found that Ni-doped ZB ZnO has a FM and an AFM state. The energy calculated results show that the FM state is more energetically favorable than its AFM state. The results show that Ni 3*d* and O 2*p* have a strong *p*-*d* hybridization effect at the Fermi level for the FM coupling state, with 100% electron-spin polarization and semi-metallic magnetic properties. For the AFM coupling state, the spin-up and spin-down orbits are symmetrically distributed near the Fermi level, and both pass through the Fermi level, with metallic magnetic properties. Moreover, we also found that in the Ni 3*d* orbital in the doping system, there exists a large exchange of splitting and crystal field splitting. The magnetic moment is mainly derived from the construction of the Ni 3*d* state, with a few parts originating from the neighboring O 2*p* state. The calculated total magnetic moment is related to the doping position of the Ni atoms. Therefore, the calculated results are useful for theoretical instruction and designing stable FM coupling and high Curie temperature of DMS.

## Figures and Tables

**Table 1:** Lattice constant of Ni-doped zinc-blende (ZB) ZnO.

Lattice Constant	Ni Position	a (Å)	b (Å)	c (Å)
Zn_32_O_32_	-	9.26	9.26	9.26
Configuration I (Zn_30_Ni_2_O_32_)	1,2	9.5304	9.5493	9.5304
Configuration II (Zn_30_Ni_2_O_32_)	1,3	9.5303	9.5303	9.5494
Configuration III (Zn_14_Ni_2_O_16_)	1,2	4.6981	9.4918	9.4863
Configuration IV (Zn_14_Ni_2_O_16_)	1,3	4.6893	9.4919	9.4939

**Table 2:** Energy, magnetic coupling, bond length, and magnetic moments of Ni-doped ZB ZnO.

Composition	Coupling	Energy (eV)	Ni–O (Å)	Zn–O (Å)	Ni1 (μ_B_)	Ni2 (μ_B_)	O (μ_B_)	Population	∆*E* (eV)
Zn_32_O_32_	-	−68072.1	-	2.003	-	-	-	0.35	-
Configuration I	AFM	−68055.4	2.045	2.060	0.77	−0.77	0.05	0.40	0.5
Configuration I	FM	−68055.9	2.053	2.061	0.79	0.79	0.11	0.41	
Configuration II	AFM	−68055.3	2.064	2.065	0.81	−0.81	0.07	0.38	0.2
Configuration II	FM	−68055.5	2.064	2.065	0.79	0.80	0.07	0.37	
